# Correlative Microscopy of Bone in Implant Osteointegration Studies

**DOI:** 10.1100/tsw.2010.223

**Published:** 2010-11-16

**Authors:** Alessandra Triré, Désirée Martini, Ester Orsini, Marco Franchi, Viviana De Pasquale, Beatrice Bacchelli, Mario Raspanti, Alessandro Ruggeri, Vittoria Ottani

**Affiliations:** ^1^ Department of Human Anatomical Sciences and Physiopathology of the Locomotor Apparatus (S.A.U. e F.A.L.), Bologna University, Italy; ^2^ Department of Human Morphology, Insubria University, Varese, Italy

**Keywords:** correlative microscopy, implant osteointegration, bone morphology

## Abstract

Routine morphological analyses usually include investigations by light microscopy (LM), scanning electron microscopy (SEM), and transmission electron microscopy (TEM). Each of these techniques provides specific information on tissue morphology and all the obtained results are then combined to give an in-depth morphological overview of the examined sample. The limitations of this traditional comparative microscopy lie in the fact that each technique requires a different experimental sample, so that many specimens are necessary and the combined results come from different samples. The present study describes a technical procedure of correlative microscopy, which allows us to examine the same bone section first by LM and then, after appropriate processing, by SEM or TEM. Thanks to the possibility of analyzing the same undecalcified bone sections both by LM and SEM, the approach described in the present study allows us to make very accurate evaluations of old/new bone morphology at the bone-implant interface.

### INTRODUCTION

The bone tissue response to implant insertion comprises a series of events culminating in an intimal apposition of bone tissue to the implant surface without the interposition of fibrous connective tissue. This process is known as osteointegration[1]. Many studies have focused on improving implant devices to enhance their biological stability and shorten healing times. For this purpose, biomolecular studies have been flanked by morphological analyses of the bone hosting the implant to yield direct information on bone quality and its response to surgical implant placement[2,3,4,5,6].

Routine morphological tests usually include histological and ultrastructural observation using light microscopy (LM), scanning electron microscopy (SEM), and transmission electron microscopy (TEM). Each of these techniques provides specific information on certain features of tissue morphology. The results of the different procedures are then combined to give an in-depth morphological overview of the examined sample. LM is the most commonly used morphological technique. Thanks to the possibility to observe samples at low magnification, it gives an overview of the examined specimens. Appropriate stainings disclose tissue types and their relative distribution. In addition, LM allows morphometric measurement of parameters defining the degree of implant osteointegration, e.g., bone-to-implant contact (BIC), bone ingrowth (BI), or bone mineralization index (BMI). SEM gives a three-dimensional view of the structures in the specimen (vessels, osteons, etc.), offering greater resolution than LM and, hence, a better view of the cell-to-cell and cell-to-implant relations with a better display of the extracellular matrix components. Lastly, TEM reveals cell morphology and activity, and the macromolecular arrangement of the matrix components. The findings yielded by these different techniques can then be combined to obtain an in-depth analysis of tissue morphology.

The limitations of traditional comparative microscopy lie in the use of different experimental samples, each intended to be processed for one specific technique, so that many specimens are required and the combined results come from different samples.

The present study describes a technical procedure of correlative microscopy that allows examination of the same sample first by LM and then, after appropriate processing, by SEM or TEM.

### MATERIALS AND METHODS

The present study used conical screw-shaped titanium implants with rough surfaces obtained by a sand-blasting and acid-etching procedure (Or-Vit, Castelmaggiore, Bologna, Italy).

Implants were inserted in tibial diaphyses of 3-year-old mongrel sheep. The area for implant insertion (medial surface of the diaphysis) was exposed, removing the periosteum, and 3.8-mm diameter holes were bored in a transverse direction to the bone surface using a low-speed drill cooled with phosphate-buffered saline (PBS). The animals were sacrificed by lethal injection at two different experimental times (4 and 12 weeks after implant insertion). Each tibia was resected and tissues surrounding the bone were removed. Cylindrical bone samples with the base perpendicular to the longitudinal bone axis, each containing a single implant, were taken from the medial region of the tibial diaphysis. All surgical procedures were performed according to Italian and European law on animal experimentation and the ethical principles stated in the “Guide for the Care and Use of Laboratory Animals”[7].

#### Light Microscopy

All samples were rinsed in 0.1 M cacodylate buffer (pH = 7.2), fixed in Karnovsky solution (4% paraformaldehyde and 2.5% glutaraldehyde in 0.1 M cacodylate buffer), dehydrated in an ascending ethanol series, defatted in xylol, and embedded in a methylmethacrylate-based resin system (Technovit 9100 New, Heraeus Kulzer, Hanau, Germany).

After polymerization, the samples were cut and ground (Sawing and Grinding System, Remet, Bologna, Italy) to obtain 100- to 150-μm-thick bone-implant sections parallel to the longitudinal implant axis. Sections were glued to plastic slides using a methylmethacrylate-based glue (Technovit 7210 VLC, Heraeus Kulzer, Hanau, Germany) and then processed for routine histological observation. They were incubated in an 0.8% toluidine blue solution containing 0.2% pyronin G, rinsed under tap water, incubated in an 0.5% acid fuchsine solution, and then observed under the light microscope (BX41, Olympus Optical Co. Europa GmbH, Germany).

All sections were deacrylated using (2-methoxyethyl)-acetate (Carlo Erba, Milan, Italy). Each slide was immersed in (2-methoxyethyl)-acetate until the bone-implant section was completely detached from its surface. The section was then immersed in a fresh solution of (2-methoxyethyl)-acetate for 20 days, renewing the solution every 4 days and periodically checking on the deacrylation process. Some deacrylated sections were processed for SEM and others for TEM.

### Scanning Electron Microscopy

Deacrylated sections were rinsed to remove (2-methoxyethyl)-acetate by immersion in 100% ethanol. The implant was gently removed from some of the sections and then all sections were immersed in hexamethyldisilazane (Sigma-Aldrich, Seelze, Germany) and dried under a hood. Dried sections were mounted on aluminum stubs using a carbon bioadhesive film, then coated in gold/palladium for observation on a Philips 515 scanning electron microscope (Philips, Eindhoven, Holland) fitted with secondary electron (SE) and backscattered electron (BSE) detectors and operated at 15 kV.

### Transmission Electron Microscopy

Deacrylated sections were rinsed in 100% ethanol and rehydrated in a descending ethanol series. After implant removal, sections were decalcified by immersion in an acidic solution of disodium EDTA (Osteodec, Bio-Optica, Milan, Italy), postfixed in 1% osmium tetroxide (Sigma-Aldrich, Seelze, Germany), dehydrated in an ascending ethanol series, and embedded in an epoxy resin (araldite). Ultrathin sections were obtained from these re-embedded samples using an ultramicrotome (Reichert OM-U3, Reichert, Depew, NY, USA) fitted with a diamond knife. Sections were contrasted with uranyl acetate and lead citrate for TEM observation (Philips CM-10 electron microscope, Philips, Eindhoven, Holland).

## RESULTS

### Light Microscopy

Sections embedded in the methylmethacrylate-based resin appeared undamaged with a good acrylic resin infiltration. Bone morphology was well preserved (Figs. 1a, 2a, 3a, 4a).

Four weeks after insertion, interwoven trabeculae of newly formed bone could be seen in the bone-implant gap. These trabeculae were clearly visible thanks to their intense fuchsine staining, and they were present on the implant surface and surrounding the pre-existing host bone surface, the latter forming chips immersed into the bone marrow. Bone trabeculae formed networks arranged around newly formed vascular structures (Figs. 1a, 3a). Twelve weeks after insertion, the bone-implant gap was filled with mature bone tissue, indicating complete implant osteointegration. Newly formed bone areas organized in osteonic structures throughout the pre-existing bone tissue were visible both proximal and distal to the implant, indicating bone rearrangement (Figs. 2a, 4a).

### Scanning Electron Microscopy

Deacrylated sections processed for SEM did not show resin residues on their surface. The lack of resin allowed a three-dimensional display of the concentric lamellae typical of osteons, and the parallel and straight orientation of interstitial lamellae. It also highlighted the vascular bundle in the Havers canals (Figs. 1b, 2b).

Each SEM image was clearly corresponding to the same area observed by LM before deacrylation (Figs. 1, 2).

The newly formed bone trabeculae, present in the bone-implant gap 4 weeks after implant insertion, surrounded numerous vascular structures and created a three-dimensional network within the bone-implant gap. SEM observation helped to disclose the morphology of bone marrow into the medullar spaces (Fig. 1b).

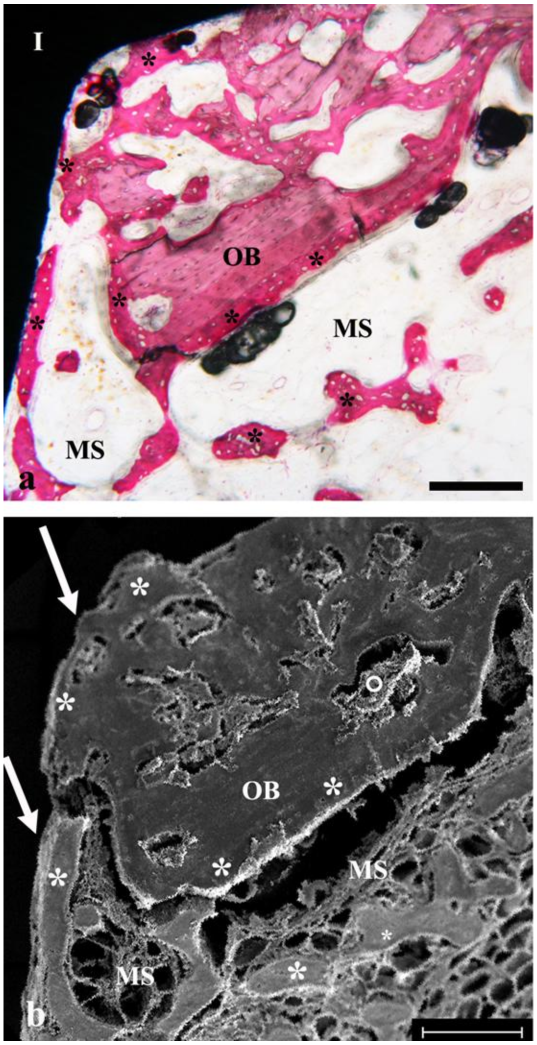

**FIGURE 1.** LM-SEM correlative analysis of the osteointegration process 4 weeks after implant insertion. (a) Section of a bone-implant sample observed by LM. Newly formed bone trabeculae (*) can be seen at the bone-implant interface, adhering both to the pre-existing bone surface and to the implant surface. I = implant; MS = medullar spaces; OB = pre-existing bone. (LM, bar = 200 μm). (b) Same section area shown in Fig. 1a after deacrylation of the section, implant removal, and bone processing for SEM. Note the bone trabeculae (*) with osteocytes in their lacunae and the medullar spaces (MS) with blood vessels (°). Implant removal allowed us to disclose the three-dimensional aspect of bone tissue at the bone-implant interface (arrows). (SEM, bar = 200 μm).

The concentric lamellar substructure of the newly formed osteons was clearly visible throughout the pre-existing bone tissue 12 weeks after implant insertion (Fig. 2b). Further structural details could be seen at the bone-implant interface. Titanium granules along the implant profile, which were not clearly distinguished in the LM images, were clearly recognizable in the corresponding backscattered electron SEM images (Fig. 2c).

### Transmission Electron Microscopy

Deacrylated LM sections processed for TEM appeared completely clear of acrylic resin residues, and the samples were well infiltrated by the epoxy resin. By carefully tracing the sections to be processed for TEM, the observed fields were correlated to well-defined areas in the corresponding LM images.

TEM images at 4 weeks disclosed the lamellar substructure of bone, characterized by a disordered arrangement of collagen fibrils in the most recently formed bone tissue (Fig. 3b) and their parallel orientation in the pre-existing bone tissue with a different orientation between two adjacent lamellae. Osteocyte lacunae and osteocyte canaliculi could also be detected (Fig. 3c).

At 12 weeks, TEM observation disclosed the different arrangement of collagen fibrils that were more disorderly dispersed in the rearranged bone areas, while they appeared parallel and tightened in the pre-existing bone areas. Collagen fibril size and period could also be clearly recognized (Fig. 4b).

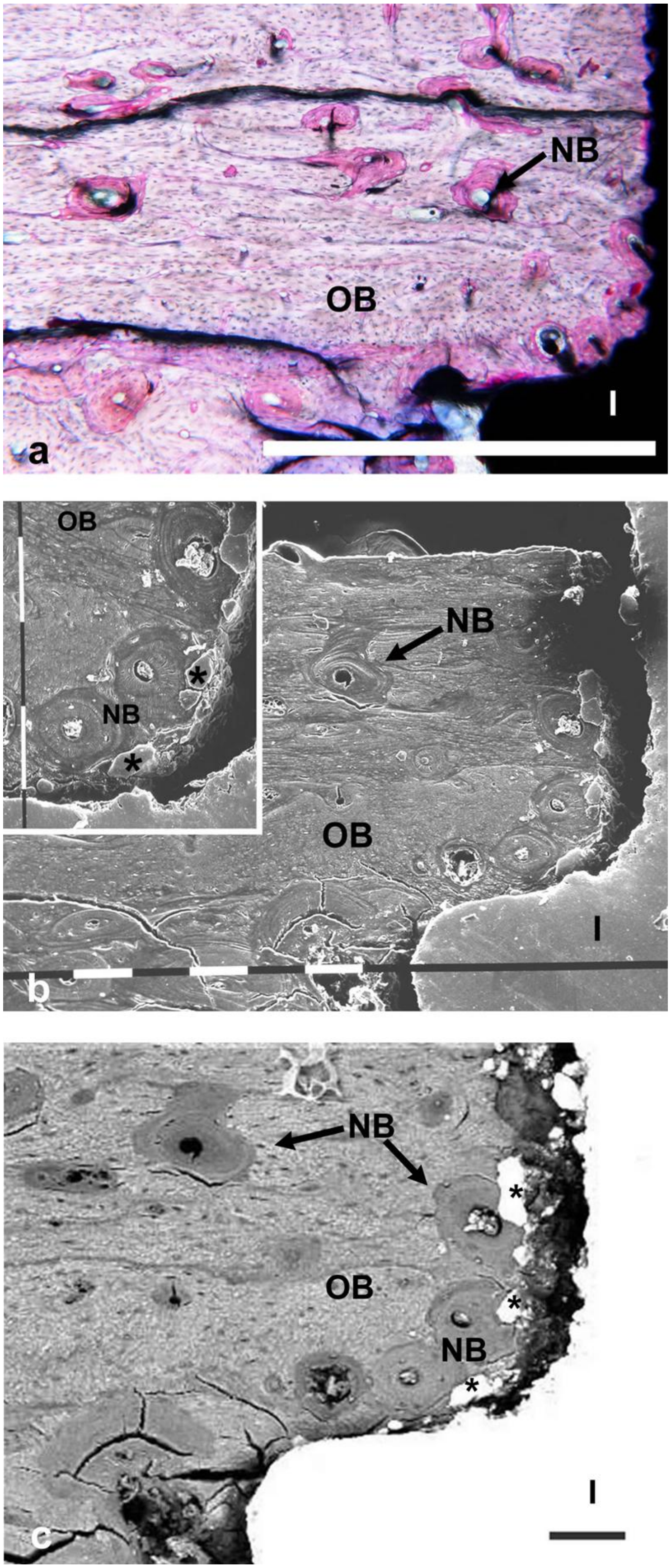

**FIGURE 2.** LM-SEM correlative analysis of the osteointegration process 12 weeks after implant insertion. (a) Section of a bone-implant sample observed by LM. Note the osteonic areas of newly formed bone tissue (NB) dispersed in the pre-existing bone (OB), both proximal and distal to the implant surface. I = implant. (LM, bar = 1000 μm). (b) Secondary electrons SEM micrograph of the same section area shown in Fig. 2a after deacrylation and processing for SEM. Note the perfect correspondence of the different elements present in the LM image. The space between the implant surface and bone surface is due to detachment of the implant during SEM processing. I = implant; NB = newly formed bone; OB = pre-existing bone. (SEM, bar = 100 μm). Insert: Detail of Fig. 2b. The newly formed bone tissue areas (NB) are clearly distinguished from the pre-existing mature bone (OB) thanks to their greater electron density and different direction of the lamellae. Note the Ti granules (*) detached from the implant surface that make the implant profile discontinuous. (SEM, bar = 100 μm). (c) Same section area shown in Fig. 2b observed by SEM in backscattered electron mode. Titanium granules (*) are clearly recognizable at the bone-implant interface. I = implant; NB = newly formed bone; OB = pre-existing bone. (SEM, bar = 100 μm).

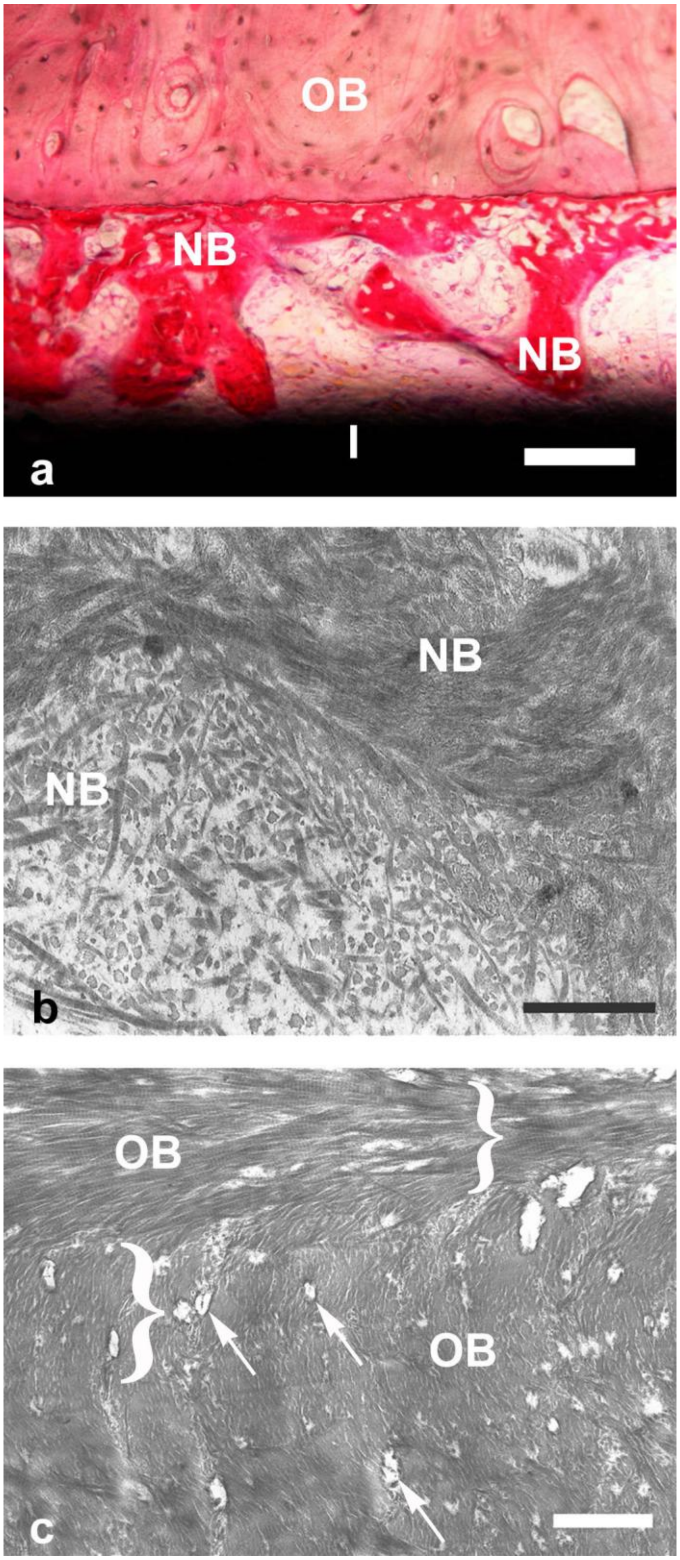

**FIGURE 3.** LM-TEM correlative analysis of the osteointegration process 4 weeks after implant insertion. (a) Section of a bone-implant sample observed by LM. Trabeculae of newly formed bone tissue (NB) can be seen at the interface forming a network within the bone-implant gap. I = implant; OB = pre-existing bone. (LM, bar = 100 µm). (b) Newly formed bone tissue (NB) at the bone-implant interface from the same section area shown in Fig. 3a after deacrylation of the section, implant removal, and bone processing for TEM. Note the organization of the more recently formed bone tissue with disorderly arranged collagen fibrils loosely distributed in the extracellular matrix. (TEM, bar = 1 µm). (c) Mature lamellar bone tissue (OB) from the same section area shown in Fig. 3a after deacrylation of the section, implant removal, and bone processing for TEM. Note the parallel orientation of collagen fibrils, strictly bundled together into bone lamellae, and their different orientation in two adjacent lamellae (brackets). Also note the osteocyte canaliculi containing cell extensions in transverse section (arrows). (TEM, bar = 1 µm).

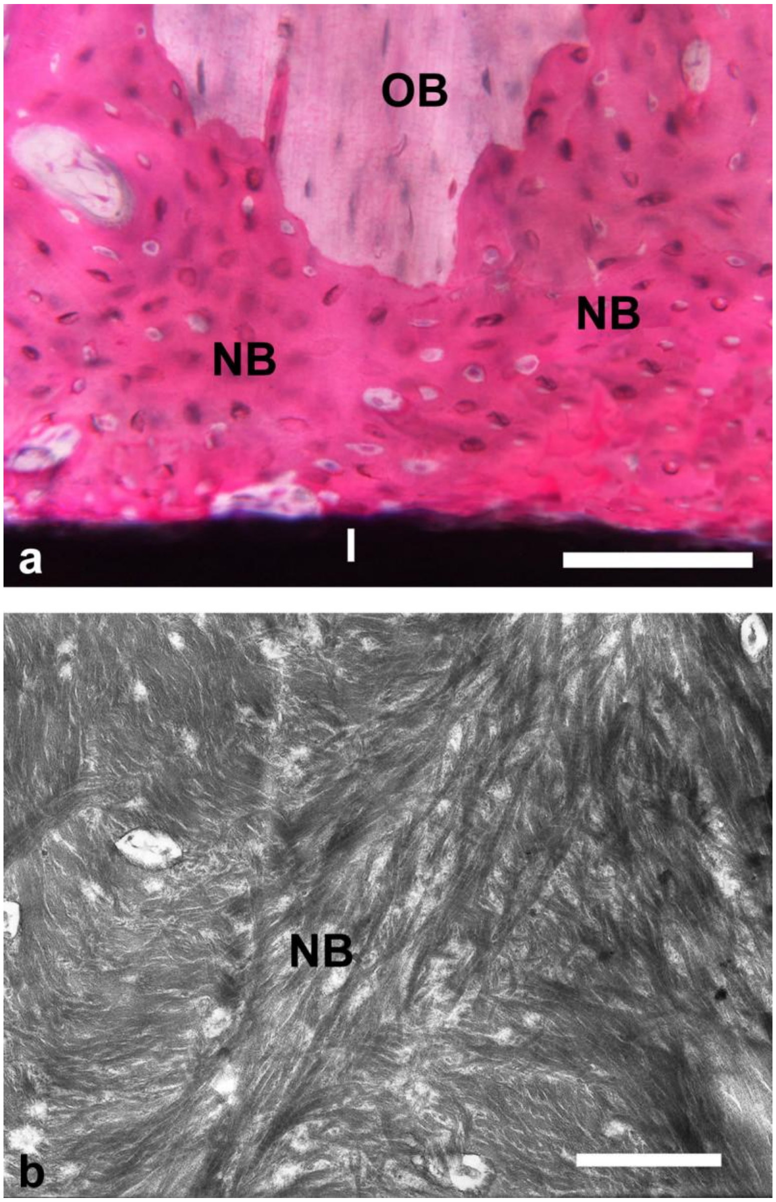

**FIGURE 4.** LM-TEM correlative analysis of the osteointegration process 12 weeks after implant insertion. (a) Section of a bone-implant sample observed by LM. Note the extensive areas of rearranged bone (NB) in contact with host bone (OB). I = implant. (LM, bar = 100 μm). (b) Detail of bone from the same section area shown in Fig. 4a after deacrylation of the section, implant removal, and bone processing for TEM. The figure displays a rearranged bone tissue area (NB) with differently oriented collagen fibrils. Osteocyte canaliculi with transversally cut cell extensions can also be detected. (TEM, bar = 2.5 μm).

### DISCUSSION AND CONCLUSIONS

One of the limitations of traditional comparative microscopy lies in the use of different samples to be processed for different morphological techniques (SEM, TEM, LM). Collating the results of each technique may disclose inconsistencies due to differences in the samples used.

In implantology, bone response to implant placement may vary widely, not only between different subjects or distant anatomical sites, but also in adjacent regions of the same host bone.

Many correlative microscopy studies have compared traditional LM (paraffin-embedded samples) and TEM by removing paraffin from the sections observed by LM and re-embedding them in epoxy resins for TEM examination[8,9]. Acrylic resins were introduced as embedding media for LM observation many years ago, and they proved to be particularly suitable for studying the osteointegration process. One of the advantages of using these resins, in fact, is the ability to process undecalcified bone samples as well as bone samples containing implants[10,11,12,13]. Procedures to remove epoxy or acrylic resins have been used in some correlative microscopy studies of soft tissues, which started with LM and TEM observations and then analyzed the same samples by SEM after resin removal[14,15,16,17]. Procedures of resin removal from bone samples were used in some LM studies, but they aimed at immunohistochemical analyses and were not related to correlative microscopy studies[12,18].

To our knowledge, no correlative LM-SEM or LM-TEM study of the osteointegration process has ever been made starting from LM observation of undecalcified bone-implant sections embedded in acrylic resins, and subsequent observation of the same samples by SEM or TEM after complete resin removal.

The correlative microscopy approach described in the present study allows a single bone section to be examined by different techniques, thereby yielding complementary information on the same histological aspects. This approach adds value to histological examination and, in some cases, drastically reduces the likelihood of misinterpretation.

This approach is particularly well suited to the study of the osteointegration of dental and orthopedic implants. It allows us to distinguish more clearly the peri-implant tissues and to make more accurate evaluations concerning the bone-implant interface. This technique may be applied to bone samples of any size, and to both trabecular and compact bone. The processing should take into account the differences between samples, since the embedding and deacrylation procedures require different time lapses depending on the bone size and type. The comparison between LM and SEM images at the bone-implant interface allows a more precise measurement of the extent of bone-to-implant contact in the examined area, while the comparison between LM and TEM provides information on bone matrix organization in a selected peri-implant area. In turn, this allows a more accurate TEM definition of the metabolic and functional state of the newly formed bone tissue. Further studies of the osteointegration process focusing on cell activity with the use of immunohistochemical analyses are already in progress.

### ACKNOWLEDGMENTS

The present study was supported by Or-Vit Srl, Castelmaggiore, Bologna, Italy.
